# MicroRNA-488 serves as a diagnostic marker for atherosclerosis and regulates the biological behavior of vascular smooth muscle cells

**DOI:** 10.1080/21655979.2021.1953212

**Published:** 2021-07-21

**Authors:** Zhen Li, Congjian Xu, Di Sun

**Affiliations:** Department of Cardiology, Shengli Oilfield Central Hospital, Shandong, China

**Keywords:** Mir-488, atherosclerosis, diagnostic value, vascular smooth muscle cell, biological behavior

## Abstract

Atherosclerosis (AS) is one of the main causes of cerebral infarction. Researches on AS mainly focus on the gene level, among which microRNA is the research hotspot nowadays. This study investigated the diagnostic value of aberrant serum miR-488 in AS patients, and further explored the effect of abnormally expressed miR-488 on the biological behavior of vascular smooth muscle (VSMCs) cells by cell transfection. The qRT-PCR was used to investigate the expression level of miR-488 in 125 AS patients and 60 healthy controls. The diagnostic value of miR-488 was analyzed by the receiver operator characteristic (ROC) curve. CCK-8 and Transwell assays were used to detect the ability of miR-488 on the proliferation and migration ability of VSMCs cells. Serum expression of miR-488 in AS patients was higher than that in healthy controls. The expression level of miR-488 was significantly positively correlated with the Carotid Intima-Media Thickness (CIMT) value. The AUC of the ROC curve was 0.892, specificity was 99.3%, and sensitivity was 77.6%. In VSMCs cells, overexpression of miR-488 significantly promoted the proliferation and migration ability. The high expression of miR-488 is a good diagnostic marker for AS. The upregulation of miR-488 promotes VSMCs cell proliferation, and migration, which may provide a new theory for the treatment of AS.

## Graphical Abstract

**Figure:**
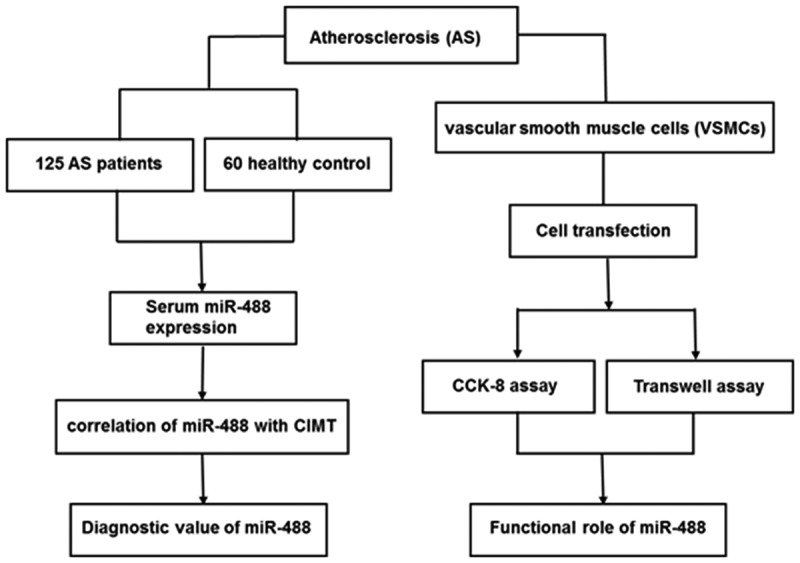

## Introduction

Atherosclerosis (AS) is a major risk factor for cardiovascular disease, it is also one of the leading fatal diseases worldwide [1,2]. AS is considered as an immune-mediated chronic inflammation of the arterial wall and its detailed pathogenesis is very complex [3,4]. The accumulation of lipids in the arterial wall is the first step in the formation of AS. Changes in lipid metabolism increase the risk of metabolic disorders in the brain [5,6]. Moreover, some patients with AS do not have any symptoms in the early stage. When AS occurs, it may cause myocardial infarction, stroke, peripheral artery disease, and other common complications. Although AS management is a benefit of statins, statins have inevitable side effects and do not meet clinical needs [7]. Therefore, a new therapeutic approach is needed to accurately diagnose and evaluate the prognosis of AS [8,9].

MicroRNAs (miRNAs) are a class of small, evolutionally conserved endogenous, non-coding, single-stranded RNA molecules about 22 nucleotides long [10,11]. Current studies demonstrated that miRNAs are closely related to the occurrence and progression of cardiovascular diseases, leukemia, diabetes, tumors, and other diseases [12,13]. In the study of miRNA and AS, it was found that miRNA is mainly involved in the regulation of the occurrence and development of carotid intima-media thickness, hemodynamics, oxidative stress, cholesterol metabolism, vascular smooth muscle cells (VSMCs), angiogenesis, inflammatory response, foam cells, and stable fibrous cap [14,15].

A recent study identified that miR-488 has an abnormally high expression in acute ischemic stroke and can predict the poor prognosis of acute ischemic stroke patients [16]. It is well known that the two main complications of AS are acute myocardial infarction and acute ischemic stroke. Since miR-488 performs an important role in an emergency stroke, we speculated that miR-488 could influence the occurrence and progression of AS. In this study, we explored the role of serum miR-488 and its diagnostic value in AS.

This study aimed to investigate whether miR-488 has diagnostic value in distinguishing AS patients from healthy individuals and whether the miR-488 expression is involved in the progression of AS. In the present study, we detected the expression of miR-488 in the serum of 60 healthy controls and 125 AS patients and evaluated the diagnostic value of miR-488 in AS patients by analyzing the clinical data of the subjects, and the regulation of vascular smooth muscle cell proliferation and migration was also investigated.

## Materials and methods

### Study population and clinical data collection

125 asymptomatic AS patients and 60 healthy controls were recruited in Shengli Oilfield Central Hospital from January 2018 to October 2019. Patients diagnosed with hemangioma, acute myocardial infarction, heart failure, hypertension, angina pectoris, stroke, and other serious cardiovascular diseases were excluded from the study population. This study was conducted with the approval of the Medical Ethics Committee of Shengli Oilfield Central Hospital and all participants signed informed consent forms. Physical examination was performed on all participants, and common carotid artery intima-media thickness (CIMT) was measured using an ATL HDI 3000 ultrasound system (Advanced Technology Laboratories, Bothell, WA, USA). The inclusion criteria for AS patients were 0.9 mm ≤ CIMT≤ 1.2 mm. Blood was collected and centrifuged to preserve the serum, then stored at −80°C for further experiment.

### Cell culture and transfection

Human VSMCs were purchased from the American Type Culture Collection (ATCC) and cultured in the Dulbecco’s modified Eagle’s medium (DMEM; Gibco, CA, USA) supplemented with 10% fetal bovine serum (FBS; PAN, Aidenbach, Germany). The miR-488 mimic (5ʹ- UUGAAAGGCUAUUUCUUGGUC-3ʹ), mimic negative control (mimic NC; 5ʹ-UUCUCCGAACGUGUCACGUTT-3ʹ), miR-488 inhibitor (5ʹ-GACCAAGAAAUAGCCUUUCAA-3ʹ), or inhibitor NC (5ʹ-CAGUACUUUUGUGUAGUACAA-3ʹ) were synthesized by RiboBio (Guangzhou, China) and transfected into cells using Lipofectamine 3000 (Invitrogen, Carlsbad, CA, USA) according to the manufacturer’s manual.

### RNA extraction and quantitative real-time PCR

TRIzol (Invitrogen, Carlsbad, CA, USA) was used to extract total RNA from cells and the serum according to the instructions. The extracted RNA was reversed-transcribed into cDNA using a One-Step PrimeScript miRNA cDNA Synthesis Kit (Takara, Tokyo, Japan). Then, qRT-PCR assay [17] was conducted to detect the miRNA expression on an ABI 7300 QRT-PCR (Applied Biosystems, Foster City, CA) instrument using the SYBR Green I Master Mix kit (Invitrogen, USA). The PCR condition was an initial denaturation of 95°C for 5 min, followed by 30 cycles of 94°C for 30 s, 60°C for 20 s, 72°C for 20 s, and a final extension at 72°C for 10 min. U6 was used as an internal control for miRNA quantification, and the relative expression quantity in the 2^−ΔΔCt^ method to calculate. The primer sequences for PCR were as follows: miR-488, 5ʹ-TGCGGCTTGAAAGGCTATT-3ʹ (forward) and 5ʹ-ATGGAGCCTGGGACGAGAC-3ʹ (reverse); U6, 5ʹ-CTCGCTTCGGCAGCACA-3ʹ (forward) and 5ʹ-AACGCTTCACGAATTTGCGT-3ʹ (reverse).

### Cell proliferation and migration assays

After 48 h of transfection, the transfected cells (2 × 10^3^ cells/well) were collected and inoculated into 96-well plates, then 10 μL CCK-8 reagent (Dojindo, Kumamoto, Japan) was added to each well [18]. The absorbance at 450 nm was measured using a microplate reader (ELx800, Bio-Tek Instruments, Winooski, VT, USA) every 24 hours, and the cell activity was observed.

Cell migration ability was determined by the Transwell assay (8 µm pore size, Corning, USA) [19]. The transfected cells (5 × 10^4^ cells/well) were inoculated into the upper chamber of the inserts in serum-free DMEM, while the lower chamber was filled with DMEM added with 10% FBS as the attractant. After incubation at 37°C for 24 h, the cells were immobilized at room temperature for 20 min. Five fields were randomly selected under an inverted microscope (Olympus Corporation, Tokyo, Japan), and counted the number of cells. The average number of migrated cells was taken as the experimental results.

### Statistical analysis

All statistical analyses of data in this study were using SPSS 21.0 software and GraphPad Prism 7, the comparison between two groups using unpaired Student’s t-test, while one-way ANOVA analysis followed by Tukey’s post hoc test was used to compare the means among three groups. ROC curve was used to evaluate the diagnostic value, and Pearson correlation coefficient analysis was used to detect the link between the two variables degree and direction. *P* < 0.05 was considered statistically significant.

## Results

In this study, serum miR-488 expression levels in AS patients and healthy controls were detected using qRT-PCR and compared the correlation with CIMT. The diagnostic value of serum miR-488 was evaluated using the ROC curve. Then, we conducted miR-488 mimics and inhibitor models in VSMCs cells and performed CCK-8 and Transwell assays to explore the functional role of miR-488 on VSMCs cell proliferation and migration.

### Clinicopathological characteristics in AS patients and healthy controls

Table 1 analyzed the demographic characteristics and clinical data in AS patients and healthy controls, it was found that the AS patients were significantly correlated with the C-reactive protein (CRP) compared with the healthy controls (P < 0.001). However, there was no obvious difference in age, gender, body mass index (BMI), total cholesterol, high-density lipoprotein cholesterol (HDL-C), low-density lipoprotein cholesterol (LDL-C), triglyceride, heart rate, systolic blood pressure (SBP), and diastolic blood pressure (DBP) (*P* > 0.05).

**Table 1.** Clinical data of the study population

Features	Healthy controls (n = 60)	AS patients (n = 125)	*P* value
Age (years)	49.62 ± 4.73	50.71 ± 6.06	0.182
Gender (male/female)	34/36	64/61	0.488
BMI (kg/m^2^)	22.87 ± 3.71	23.24 ± 2.68	0.490
Total cholesterol (mg/dl)	167.78 ± 33.99	171.97 ± 23.08	0.390
HDL-C (mg/dl)	51.49 ± 10.01	52.95 ± 8.07	0.326
LDL-C (mg/dl)	118.46 ± 20.41	120.90 ± 16.27	0.419
Triglyceride (mg/dl)	169.93 ± 31.54	177.85 ± 20.97	0.081
Heart rate (beats/min)	78.36 ± 18.68	81.91 ± 15.37	0.173
SBP (mm Hg)	131.62 ± 14.99	129.54 ± 10.66	0.339
DBP (mm Hg)	78.68 ± 5.26	78.23 ± 8.96	0.669
CRP (mg/l)	6.21 ± 1.60	17.16 ± 2.47	0.000

### The expression level of serum miR-488 in AS patients and the correlation study between miR-488 expression and CIMT

**Figure 1.**
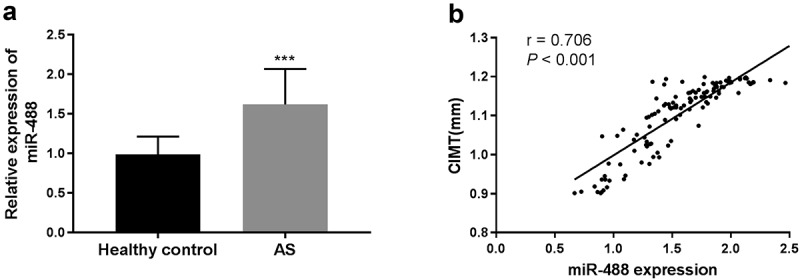
The expression level of serum miR-488 in AS patients and the correlation study between miR-488 expression and CIMT. (a) The expression level of serum miR-488 was upregulated in AS patients compared with healthy controls. ***P < 0.001. (b) Serum miR-488 level was positively correlated with CIMT in patients in AS patients (r = 0.706, ***P < 0.001)

The expression levels of miR-488 in AS patients and healthy controls were investigated using qRT-PCR. The results showed that the expression of miR-488 in AS patients was significantly higher than in healthy controls (P < 0.001, Figure 1(a)). CIMT was a subclinical indicator of AS patients independent of age, gender, and cardiovascular risk factors, and was widely used in the early detection of AS. Figure 1(b) showed the correlation of miR-488 with CIMT in AS patients, the results showed that serum miR-488 level showed a positive correlation with CIMT (r = 0.706, P < 0.001) in AS patients.

### Diagnostic value of miR-488 for AS

**Figure 2.**
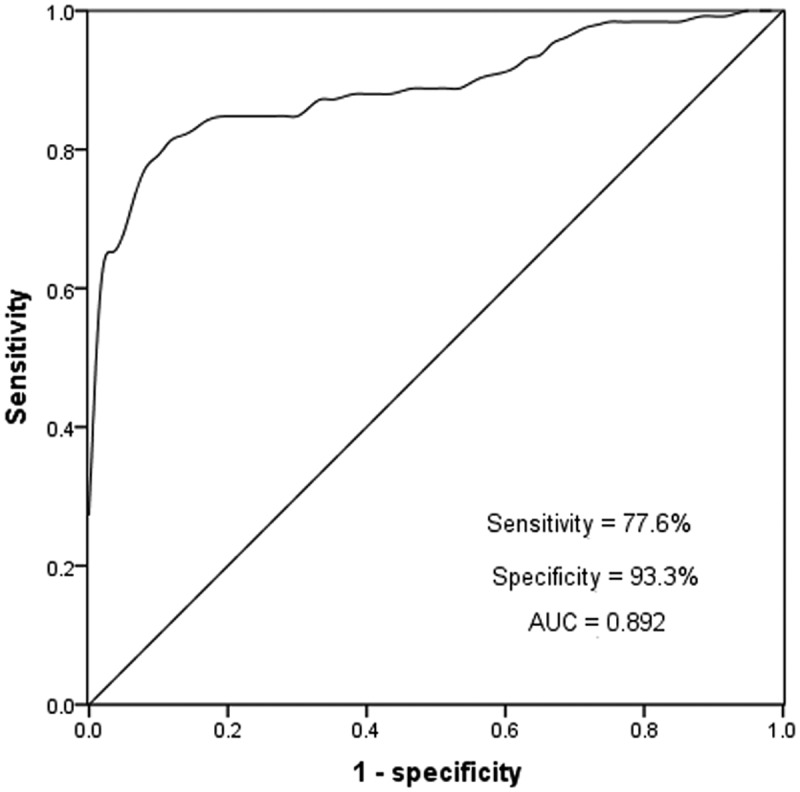
A ROC curve was established to assess the specificity and sensitivity of miR-488 to differentiate between AS and healthy controls, the miR-488 has quite well performance with an AUC score of 0.892

ROC curve is often used to evaluate the diagnostic ability of biomarkers, providing sensitivity and specificity of biomarkers. In this study, the diagnostic value of miR-488 in AS patients was evaluated by the ROC curve. As shown in Figure 2, the cutoff value of ROC was 1.30, the AUC under the curve was 0.892, the sensitivity was 77.6%, and the specificity was 99.3%. The evaluation results indicated that miR-488 had a high diagnostic value in AS patients.

### Upregulation of miR-488 promotes cell proliferation and migration of VSMCs

**Figure 3.**
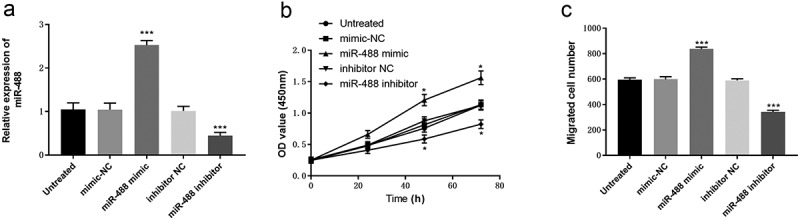
Effects of miR-488 expression levels on cell proliferation and migration abilities in VSMCs cells. (a) qRT-PCR was used to analyze the expression level of miR-488 after transient transfection with miR-488 mimic/inhibitor (or mimic/inhibitor NC). (b) The CCK-8 assay was performed to study cell proliferation. (c) Cell migration abilities were assessed with Transwell assay. ***P < 0.001, *P < 0.05

VSMCs were the only cell type in the middle layer of the blood vessel wall. AS is closely related to the proliferation and migration of VSMCs. Figure 3 investigated the effect of miR-488 on cell proliferation and migration in VSMCs. VSMCs cells were transfected with miR-488 mimic, mimic NC, miR-488 inhibitor, or inhibitor NC. The qRT-PCR results show that miR-488 mimic could significantly increase the expression of miR-488, while miR-488 inhibitor decreased the expression of miR-488 (P < 0.001, Figure 3(a)). The CCK-8 assay showed that overexpression of miR-488 significantly increased the proliferation ability of VSMCs cells, while the low expression of miR-488 inhibited VSMCs cell proliferation compared with the blank control group and NC group (P < 0.05, Figure 3(b)). Transwell assay was used to detect the effect of miR-488 on the migration ability of VAMCs cells. As shown in Figure 3(c), overexpression of miR-488 significantly increased the number of migrated VSMCs cells, and downregulation of miR-488 decreased the number of migrated VSMCs cells (*P* < 0.001).

## Discussion

AS is a chronic systemic inflammatory disease, and it is also the most common death disease in the world. The death toll is more than that of all cancers combined [20,21]. It is the main pathological basis of ischemic cardiovascular and cerebrovascular diseases such as coronary heart disease, cerebrovascular disease, and thromboembolic disease [22]. Up to now, the pathogenesis of AS has not been fully understood, and there are many theories involving many risk factors, leading to a lack of effective prevention and treatment drugs in clinical practice [23,24]. Therefore, it is crucial to predict the AS and take effective intervention measures at the appropriate time.

miRNAs are involved in multiple processes that lead to the development of human diseases [25,26]. miRNAs can upregulate or downregulate the expression of atherosclerosis-related genes, regulate the transduction of multiple signaling pathways in cells, and participate in the regulation of carotid intima-media thickness, hemodynamics, oxidative stress, cholesterol metabolism, vascular smooth muscle, angiogenesis, inflammatory response, foam cells, and stable fibrous cap [27,28]. In AS, the dysregulation of miRNAs has been widely reported, such as miR-155 was induced to transfer from smooth muscle cells to endothelial cells, which destroys the tight junction of endothelial cells and the integrity of endothelial barrier, resulting in increased endothelial permeability and progression of AS [29]. Macrophages play a crucial role in the pathogenesis of AS. Yanyong Xu and coworkers outline a central role of miR-34a in regulating macrophage cholesterol efflux, inflammation, and AS, suggesting that miR-34a is a promising target for the treatment of cardiometabolic diseases [30]. Interestingly, we found that miR-488 was significantly increased in patients with AS compared with healthy control, which was consistent with the expression level of acute ischemic stroke with AS complications. In acute ischemic stroke [16], the expression of miR-488 in the peripheral blood of the case group was significantly higher than that of the control group. This supports our guess that miR-488 may play an important role in AS and promote the progression of AS.

AS is considered to be a multi-factor disease caused by an inflammatory response, and the inflammatory process contributes to the occurrence of acute AS thrombosis events. CRP is an acute protein whose concentration in serum reflects the inflammation of patients and is related to cardiovascular diseases, such as AS, hypertension, myocardial infarction. In our study, CRP levels were significantly increased in patients with AS compared to healthy controls. CIMT is a tool for early detection and tracking of AS, it is increasingly favored by doctors due to its advantages of real-time, convenience, rapid, safe, and noninvasive. The increase of CIMT is considered an important indicator of subclinical AS, and our study shows that CIMT is significantly positively correlated with the expression of miR-488. It was confirmed again that AS was closely related to the high expression of miR-488. The ROC curve results showed that miR-488 was a good diagnostic marker of AS with high sensitivity and specificity. Therefore, in this study, the abnormally high expression of miR-488 was considered as a new potential marker for the diagnosis of AS.

The migration and proliferation of VSMCs into arterial intima are involved in the progress of atherosclerosis [31]. There were studies have shown that miRNAs involved in regulating the migration, differentiation, and proliferation of VSMCs may become new targets for the prevention and treatment of atherosclerosis [32]. A recent study indicated that miR-146b-3p weakened the proliferation, migration, and phenotype switch of VSMCs by targeting phosphoinositide-3 kinase catalytic subunit gamma (PIK3CG) [33]. These studies revealed the crucial role of miRNAs in AS and the regulation function of VSMCs. Therefore, it is very necessary to investigate the effect of abnormally expressed miR-488 on the biological behavior of VSMCs cells by cell transfection. CCK-8 and Transwell assays proved that overexpression of miR-488 could promote cell proliferation and migration; however, the low expression of miR-488 led to the opposite results. It seems reasonable to speculate that miR-488 may be an AS promoter and the overexpression of miR-488 may promote AS progression.

A recent study indicated that blood miR-488 expression was high in acute ischemic stroke patients and predicted poor prognosis of the patients with transient receptor potential cation channel subfamily C member 6 (TRPC6) as the target gene [16]. Combined with the present results, we speculate that miR-488 may promote the AS progression by targeting TRPC6. Although a series of experiments have proved that miR-488 may be a potential diagnostic marker of AS, the mechanism of miR-488 in the occurrence and development of AS is still unclear, and further research is needed.

## Conclusion

In summary, a series of experiments have proved in this study that miR-488 is highly expressed in patients with AS, which may be a new potential biomarker for the diagnosis of AS. Meanwhile, downregulation of miR-488 can inhibit VSMCs cells proliferation and migration, which may be a new theoretical basis and direction for more effective treatment of AS.

## Data Availability

Corresponding authors may provide data and materials.

## Highlights

- The serum expression of miR-488 is upregulated in atherosclerosis patients.
- Serum miR-488 expression has diagnostic value for AS patients.
- miR-488 promotes VSMCs cell proliferation and migration.
- miR-488 may be a novel therapeutic strategy for AS patients.
